# Unequal evolutionary conservation of human protein interactions in interologous networks

**DOI:** 10.1186/gb-2007-8-5-r95

**Published:** 2007-05-29

**Authors:** Kevin R Brown, Igor Jurisica

**Affiliations:** 1 Department of Medical Biophysics, University of Toronto, Toronto, Canada M5G 1L7; 2 Ontario Cancer Institute, Toronto Medical Discovery Tower, Toronto, Canada M5G 1L7; 3 Department of Computer Science, University of Toronto, Toronto, Canada M5G 1L71

## Abstract

**Background:**

Protein-protein interaction (PPI) networks have been transferred between organisms using interologs, allowing model organisms to supplement the interactomes of higher eukaryotes. However, the conservation of various network components has not been fully explored. Unequal conservation of certain network components may limit the ability to fully expand the target interactomes using interologs.

**Results:**

In this study, we transfer high quality human interactions to lower eukaryotes, and examine the evolutionary conservation of individual network components. When human proteins are mapped to yeast, we find a strong positive correlation (r = 0.50, *P *= 3.9 × 10^-4^) between evolutionary conservation and the number of interacting proteins, which is also found when mapped to other model organisms. Examining overlapping PPI networks, Gene Ontology (GO) terms, and gene expression data, we are able to demonstrate that protein complexes are conserved preferentially, compared to transient interactions in the network. Despite the preferential conservation of complexes, and the fact that the human interactome comprises an abundance of transient interactions, we demonstrate how transferring human PPIs to yeast augments this well-studied protein interaction network, using the coatomer complex and replisome as examples.

**Conclusion:**

Human proteins, like yeast proteins, show a correlation between the number of interacting partners and evolutionary conservation. The preferential conservation of proteins with higher degree leads to enrichment in protein complexes when interactions are transferred between organisms using interologs.

## Abstract 2

The conservation of protein-protein interaction networks can be examined by mapping human proteins to yeast and other model organisms, revealing that protein complexes are preferentially conserved, and that such conservation can yield biological insights.

## Background

The evolution of high-throughput (HTP) technologies in the post-genomics era has taken scientists from the characterization of single proteins to the investigation of entire interactomes. Biological techniques have been supplemented with *in silico *approaches to map interactomes between species using orthologs, making predictions about new interactions that have not yet been demonstrated experimentally. This concept of interologs was first proposed by Matthews *et al*. [1] to transfer yeast protein-protein interactions (PPIs) to worm; however, only 16% to 31% of the interactions that were predicted were validated by yeast two-hybrid (Y2H) assay. Possible explanations for this modest result include technical aspects of the Y2H assay, predictions from false positive PPIs, or the lack of interaction conservation between species that are distant by more the 900 million years. Another study using interactions predicted from multiple organisms have found greater conservation of interologs (50% to 100%), suggesting that higher quality sources can improve the experimental validation [2,3]. Finally, Yu *et al*. [4] found that identifying interologs by a reciprocal best-hit approach (RBH; see Materials and methods) had a 54% true-positive rate, which was higher than both the method used by Matthews *et al*., and the generalized interolog approach.

A combination of low-throughput (LTP) and HTP interaction studies have produced large networks of interacting proteins in *Homo sapiens *(human), *Rattus norvegicus *(rat), *Mus musculus *(mouse), *Drosophila melanogaster *(fly), *Caenorhabditis elegans *(worm), and *Saccharomyces cerevisiae *(yeast) (see Additional data file 1 for sources). In addition, manual curation of the scientific literature has resulted in large PPI databases in machine readable format [5-9]. These resources have been supplemented by several groups, leading to PPI databases using interologous prediction of human interactions from model organisms [10-12], some of which integrated predicted, curated, and experimentally derived interactions [10,13].

Analyses of these large datasets revealed interesting characteristics within interactomes. First, co-expressed genes encode proteins that are more likely to interact than randomly selected proteins [14,15]. Additionally, stable complexes show a much higher level of co-expression than transient complexes [16,17], as well as higher co-localization. Furthermore, it was determined that highly connected proteins ('hubs') can be subdivided into two classes: 'party' hubs, which interact simultaneously with multiple partners; and 'date' hubs, which interact at different times and places [18] based on the degree of co-expression. This agrees with the analysis of Jansen *et al*. [16], as party hubs are found within large stable complexes such as the 26S proteasome, which show a high degree of gene co-expression.

Analysis of the yeast PPI networks has revealed that not all interacting proteins display the same rate of evolutionary conservation; higher degree proteins tend to display a slower rate of evolution [19,20], and thus are more conserved [21]. Additionally, higher modularity in the PPI network is associated with an increased evolutionary retention rate [21-23]. Taken together, this suggests that highly interconnected hub proteins, such as those found in stable complexes, are more conserved evolutionarily. This was confirmed by Mintseris and Weng [24], who found that stable interacting proteins have greater conservation of the amino acid residues in the interaction interfaces than transient ones.

In light of the differences in conservation of the proteins that comprise the interactomes, it is important to re-examine the conservation of interologous interactions across species. We expect more highly connected proteins to be preferentially conserved, particularly those from highly interconnected complexes. Thus, we expect increased conservation of stable complexes across species. However, the effect of evolutionary distance on conservation has not yet been established, nor how the preferential conservation of large complexes affects the interologous transfer of networks between organisms.

While the previous work was carried out on yeast PPI networks, little is known about the properties of the human interactome. Using the known human interactome (that is, literature-based interactions from BIND, BioGrid, DIP, HPRD, and MINT, plus HTP experiments; see Additional data file 1) as a starting point, we created interologous networks in multiple organisms (see Additional data file 2) [25]. The evolutionary distance between yeast and any of the other five organisms under consideration falls between 990 million and 1.5 billion years. Fine detail in the changes in the networks may be difficult to observe over such large distances. However, with a growing human PPI dataset (currently 33,713 known unique PPIs) we can compare it to mouse/rat (91 million years), fly/worm (990 million years), and yeast (1.5 billion years) [26,27]. This resource enables us for the first time to evaluate the changes in predicted interaction networks over evolutionary distance.

From the above it follows that the evolutionary conservation of PPIs across organisms is not uniform. Therefore, we examined the networks that are transferred between organisms for the preferential conservation of protein complexes, and the rate of PPI conservation as a function of evolutionary distance. We find that human proteins display a similar evolutionary relationship as yeast proteins, with higher degree proteins being conserved preferentially. Additionally, as the evolutionary distance between organisms grows, the preferential conservation of interologs within stable complexes increases.

## Results

### Properties of PPI networks

**Figure 1:**
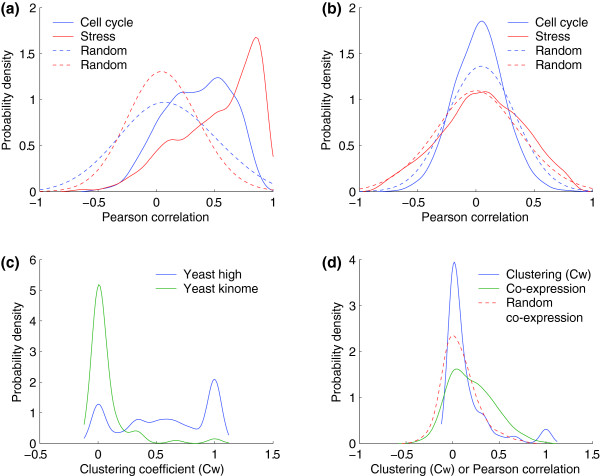
Properties of PPI networks. **(a) **Co-expression of yeast 'high confidence' protein interactions (solid lines) and random protein pairs (dotted lines) using two microarray datasets. This network is enriched in stable complexes, represented by a high mean correlation. **(b) **Co-expression of the yeast 'kinome' [31], which is enriched for transient interactions. This type of interaction shows co-expression that is highly similar to the random distribution (dotted lines). **(c) **Distribution of clustering coefficients in stable and transient PPI networks. Complexes are represented by a high C_w _(blue line), while the sparsely connected transient network is typified by a low C_w _(green line). **(d) **The properties of the human interaction network. The clustering coefficients indicate that this network is more sparsely connected, with few protein complexes. The co-expression profile is only slightly higher than the randomly generated distribution, suggesting the presence of many transient PPIs.

In order to characterize aspects of the predicted interaction networks we must first establish the properties of interest. In particular, we are interested in the conservation of stable complexes versus transient interactions, and thus we need to be able to distinguish between them. Stable complexes are highly interconnected (high clustering coefficient, C_w_), and show a high degree of co-expression. As an example of a network highly enriched in protein complexes, we examined the yeast 'high confidence' dataset from von Mering *et al*. [28]. This dataset comprises interactions determined by multiple experimental datasets and techniques. Using two independent microarray datasets [29,30], we observed much higher than random gene co-expression (Figure 1a), which demonstrates the abundance of stable complexes. A comparable network that is enriched in transient protein interactions is the yeast 'kinome', which is based on kinase-substrate interactions [31]. In contrast, the transient interactions (Figure 1b) are indistinguishable by gene co-expression from the random protein pairs. The large number of complexes in the yeast 'high confidence' dataset is also characterized by the overabundance of highly clustered proteins (Figure 1c, blue curve; Additional data file 3), while the transient PPI dataset shows almost no clustering (Figure 1c, green curve). The human PPI network was examined to assess whether it more closely resembles the high confidence or kinome datasets (Figure 1d). There are a small number of highly clustered proteins, with the majority showing little or no clustering, akin to the transient yeast kinome. Similarly, the gene co-expression is only slightly higher than random as it was for the yeast kinome, which suggests a dominant presence of transient interactions within this network.

### Interactome datasets

We have integrated known, experimental and predicted PPIs for five model organisms and human in the OPHID database [10]. The properties of these networks are listed in Table 1. In particular, there are 33,713 known unique PPIs in the human network, with a mean degree of 6.85 and a mean C_w _(<C_w_>) of 0.1453. The yeast protein interaction network, which has been built primarily through extensive HTP studies, comprises 95,104 unique PPIs, with both a mean degree (<k>) and <C_w_> that is much higher than the human network, at 33.61 and 0.2622, respectively. The high clustering in this network is reflective of an abundance of protein complexes obtained by large-scale mass spectrometry experiments [32-34]. Worm, fly, mouse and rat PPI networks have also been compiled, and can be integrated with predicted interactions, or used to predict interologous interactions in other organisms. The properties of these networks are also summarized in Table 1.

**Table 1:** Characteristics of known PPI networks for each source organism

Organism*	PPIs	Proteins	<k>	C_w_
Human	33,713	9,799	6.85	0.1453
Rat	653	538	2.43	0.1357
Mouse	1,810	1,674	2.16	0.1581
Fly	24,688	7,549	6.52	0.0245
Worm	5,611	3,230	3.46	0.1333
Yeast	95,104	5,652	33.61	0.2622

*See Additional data file 1 for a list of data sources.

### Construction of interologous networks

PPI networks were transferred between organisms using interologs. Briefly, interactions from organism X are inferred in organism Y if the two interacting proteins from X have orthologs in Y. Applying the same approach as we used for OPHID [10], we generated a database of orthologs between each of the six organisms of interest. Orthologs are then used to map the interactome of one organism into another.

Yu *et al*. [4] examined the conservation of interologs using several metrics. One such metric is the joint sequence identity, which is defined as the geometric mean of the percent identities of the two orthologs involved in the predicted interaction. In general, Yu *et al*. found the conservation of interologs increased markedly above a joint identity of 40%, up to 100% conservation at a threshold of 80% identity. We computed the joint sequence identity for all interologs transferred from the human network, and the cumulative distributions are shown in Additional data file 4. It is interesting to note that the cumulative distributions are shifted according to the evolutionary distance, with the predicted yeast interactions having the lowest joint identity distribution, and the rat and mouse having the highest. More importantly, nearly 50% of the yeast interologs have a joint sequence identity greater than 40%. Even higher conservation was observed for the worm and fly interologs (52% and 70% of interologs, respectively), while 99.9% of the mouse and rat interologs were above 40% identity. While a high joint sequence identity does not guarantee conservation of the mapped interolog, it does suggest an increased probability of the interaction being conserved between species.

**Figure 2:**
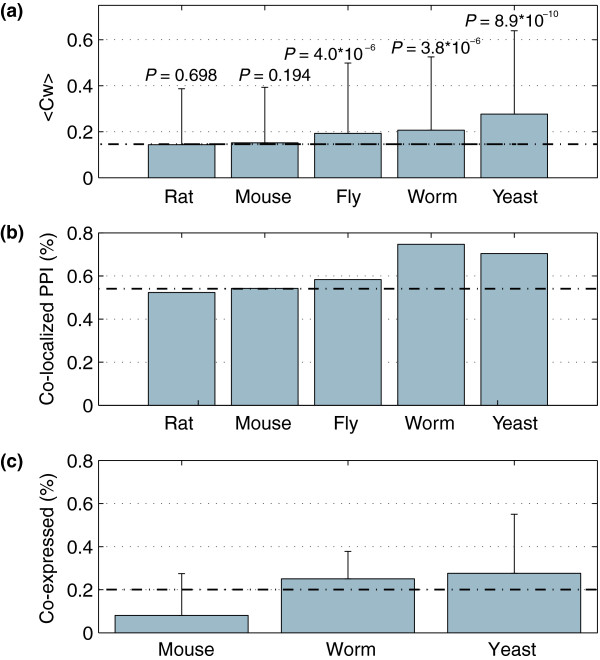
Effect of interolog transfer across evolutionary distance. Interologous protein interactions were predicted from the known human PPI network. **(a) **The mean C_w _for the predicted network in each model organism (mean ± standard deviation), averaged over all nodes with k > 1. *P *values indicate the significance of the difference from the human interactome. **(b) **The mean co-localization for each model organism network is shown, normalized against the number of PPIs with localization data for both proteins. **(c) **The Pearson correlation of genes encoding interacting proteins in each organism (mean ± standard deviation). In all cases, the average correlation is significantly higher than a randomized network (*P *<< 0.001). In each plot, the dotted line indicates the average level for the human network.

Table 2 summarizes the characteristics of the human interactome as it is transferred into each of the five lower eukaryotes. These data show that the number of interactions predicted decreases as the evolutionary distance increases. This can be attributed to both fewer orthologs being found between more distant organisms as well as the fact that the more distant organisms in this study have smaller proteomes. Interestingly, <C_w_> is increasing in the interologous networks (Figure 2a), while <k> is decreasing. The rise in C_w _indicates that the interologous networks are more highly interconnected than the original human network. In general, this increasing density results from low degree nodes (k < 4) being lost through the interolog mapping, while nodes with degrees ranging from 5 to 40 are preferentially conserved (*P *< 0.05, Fisher's exact test). For clarity, this does not imply any structural changes in the predicted networks, but rather that some of the sparsely connected interactions are being 'filtered out' through the interolog prediction method. Similar trends are observed when the rat and mouse interactomes are transferred to lower eukaryotes (Additional data file 2).

**Table 2:** Characteristics of interologous interactomes predicted from human

Target organism	Predicted PPIs	Overlap*	C_w_	<k>
Human	-	-	-	-
Rat	10,597	231	0.1434	5.52
Mouse	23,251	634	0.151	6.82
Fly	2,883	93	0.1914	3.53
Worm	*2,092*	176	0.205	3.46
Yeast	750	345	0.2738	2.51

*Overlapping with known PPIs in each organism. See Additional data file 2 for characteristics of all predicted networks.

### Increased conservation by degree

**Figure 3:**
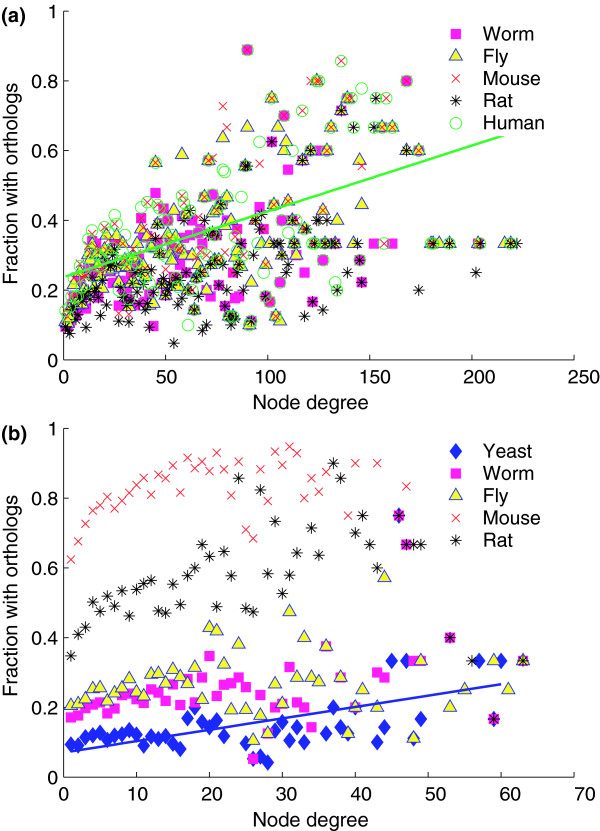
Conservation of interacting proteins by degree. **(a) **Each protein in the yeast interaction network was examined for orthologous proteins in the five higher eukaryotes, and binned according to degree. The proportion of each bin with orthologous proteins is shown. The linear trend shows the strong positive correlation (Spearman's rank *r *= 0.52, *P *= 2.8 × 10^-11^) between yeast and human proteins. **(b) **The proteins in the human interactome were compared against all five lower eukaryotes, and binned according to degree. This trendline also shows a strong correlation against yeast (Spearman's rank *r *= 0.50, *P *= 3.9 × 10^-4^), which is similar for worm and rat, and there is a weak (non-significant) correlation to fly. There was a weak negative correlation in mouse (Spearman's rank *r *= -0.02); however, the overall conservation was high, likely biasing this measurement.

Previous analysis of the yeast interactome revealed that proteins with higher degree display greater evolutionary conservation [19], although there has been some debate about this finding [20,35]. Therefore, to confirm that this relationship could be obtained using our sets of PPIs and orthologs, the fraction of yeast proteins conserved in higher eukaryotes was analyzed as a function of node degree. The relationship is indeed confirmed in Figure 3a, which shows a positive correlation between degree and conservation in higher eukaryotes (Spearman's rank *r *= 0.52, *P *= 2.8 × 10^-11^). Similar correlations are observed between yeast and worm (*r *= 0.55), fly (*r *= 0.62), mouse (*r *= 0.58), and rat (*r *= 0.58). This relationship is observed over great evolutionary distances, from 990 million years (worm/fly) to 1.5 billion years (mouse/rat/human).

Next, we examined whether human proteins display similar conservation across evolutionary distance as the yeast proteins. The most closely related species to humans in this study are mice and rats, which are only 91 million years distant, thereby providing an intermediate distance missing in the yeast comparisons. Figure 3b indicates that human proteins, in general, show increased evolutionary retention as a function of degree when mapped to yeast (Spearman's rank *r *= 0.50, *P *= 3.9 × 10^-4^), confirming that human proteins exhibit the same relationship between evolutionary distance and degree as yeast proteins. A similarly strong relationship is found between human and worm (*r *= 0.51, *P *= 2.0 × 10^-4^), and human and rat (*r *= 0.46, *P *= 4.4 × 10^-4^). A weaker (non-significant) correlation is observed between human proteins and fly (*r *= 0.17, *P *= 0.23), although it is unclear why this correlation is lower than that of the worm. No correlation is observed between human and mouse proteins as a function of degree (*r *= -0.02, *P *= 0.88), although the relationship may be affected by the uniformly high conservation seen between human and mouse proteins (the lowest conservation of human proteins in mice is 62%, observed for proteins with degree = 1).

It is also interesting to note that the data in Figure 3b stratify according to the evolutionary distance between organisms, where the mouse and rat show the greatest conservation of human proteins overall, followed by fly, worm, and finally yeast. This helps to explain the decreased number of conserved PPIs with the increased evolutionary distance in our interolog networks. Looking across the entire range of protein degrees, an average of 81% of the human proteins are conserved in mice - a number that increases with increasing degree. Similarly, on average, 59% of the human proteins are conserved in rats. As the evolutionary distance increases ten-fold (to 990 million years), the conservation rate drops to a mean of 28% in the worm and fly. Finally, on average, only 16% of the human proteins are conserved in yeast.

### Conservation of complexes

The higher degree proteins are more conserved, and the average clustering of the network increases with the increased evolutionary distance between organisms. These results suggest that complexes are more highly conserved in the interolog networks relative to other network components. We therefore considered other properties of the PPI networks that may help support this assertion, such as co-localization, and gene co-expression.

Protein complexes have been shown to display increased co-localization when compared to transient protein interactions, as judged by Gene Ontology (GO) annotations [17]. Logically, proteins must be co-localized in order to physically interact. In practice, the annotation of protein sub-cellular localization is less than complete, and stringent computational techniques must be used to avoid detecting co-localization based on generic annotations. In our analysis, 48.1% of all experimentally derived yeast PPIs are co-localized, which is similar in the worm (60.4%), fly (41.6%), mouse (65.6%), rat (43.1%) and human (54.1%). For comparison, datasets enriched in protein complexes show a much higher level of co-localization; 85.7% of the 'high confidence' PPIs (*n *= 1,601) from von Mering *et al*. [28] are co-localized, as are 88.3% (*n *= 6,705) of a yeast TAP tagging dataset [36]. In contrast, transient interactions exhibit much lower co-localization, with 36.4% of the transient kinase-substrate interactions in the yeast 'kinome' [31] co-localized.

When the human PPI network is transferred to rat or mouse, there is little change in the level of co-localization, primarily due to high conservation between the three species. However, when the human PPIs are transferred to the more distantly related fly, worm, or yeast, the level of co-localization increases (Figure 2b). In the fly, 58.3% are co-localized, while 74.7 and 70.4% of the worm and yeast interactions are co-localized, respectively. In all cases, the percentage of co-localized proteins was normalized against the number of interactions where both proteins have localization data in order to control for differences in protein annotation in each organism. Permutation testing was performed to ensure that the degree of co-localization observed in the known and predicted networks could not be obtained by random chance, and was not due to biases in sampling or annotation differences (see Additional data file 5). The increased co-localization of predicted networks in the distantly related organisms, which is higher than the source human network, experimentally derived networks, and randomly chosen protein pairs, suggests that the predicted networks are enriched for complexes relative to the original human network.

Similarly, interacting proteins within complexes should display higher gene co-expression, and thus enrichment for complexes should be apparent by comparing the mean gene co-expression of the mapped networks. Figure 2c shows that both worm and yeast display increased gene co-expression compared to humans. However, this trend is not seen in mouse, and the overall increase was not as high as we had expected. Comparisons between measurements of co-expression in different organisms may be complicated by the types of tissues used for the microarray measurements, heterogeneity in tissues or cell cycle stages, and other experimental factors from the gene expression data. Despite these challenges, our results suggest that stable protein interactions moderately increase with the evolutionary distance.

### Enrichment in detecting stable complexes

In expanding the known human PPI network with interologous predictions, we noted an increased level of gene co-expression in PPIs that were mapped from model organisms using the GeneAtlas gene expression data [37] (Figure 2c). Table 3 shows that the human interactome has a mean co-expression value of 0.241, while known human PPIs that have interologous interactions in model organisms show a mean co-expression nearly two-fold higher. This increased even further when we compared PPIs with interologous interactions in more than one model organism. When we examined PPIs conserved across three organisms, we found a mean co-expression of 0.717. Manual inspection of these interactions revealed enrichment for stable complexes such as the 26S proteasome, 40S and 60S ribosomal proteins, eIF-2 complexes, the origin recognition complex (ORC) and minichromosome maintenance (MCM) complexes, among others. This suggests that interactions detected in multiple interaction screens, observed in multiple organisms, and conserved across organisms, primarily form stable complexes. von Mering *et al*. found the yeast interactome to be enriched for ancient, evolutionarily conserved proteins [28], and it is likely that this is also true in other interaction detection screens, which would contribute to an abundance of stable, conserved complexes.

**Table 3:** Gene co-expression in known and predicted human PPI networks

Dataset	Mean correlation	n
Known human	0.241	5,201
Predicted, overlapping	0.408	242
Predicted, non-overlapping	0.412	4,571
Predicted, >1 org	0.717	115
Random	0.09	10,000

Gene expression analysis was performed on the human GeneAtlas [37]. 'Predicted, overlapping' are interactions predicted from model organisms, and also found in the known human dataset. 'Predicted, non-overlapping' are novel predictions not found in the known human interaction databases. 'Predicted, >1 org' are PPIs inferred from more than one model organism, regardless of overlap with the known human PPI network.

### Novel yeast interactions

One of the possible explanations for the low fraction of interologous predictions that were validated in Matthews *et al*. [1] is the quality of the earlier Y2H protein interactions upon which the predictions were based. In the current study, the human interactome has largely been compiled from LTP studies in the literature, which is often cited as a 'gold standard'. Interestingly, when we transfer the human interactome to yeast, 46% (345) of the predictions overlap with known yeast interactions. This is already much higher than the number validated in Matthews *et al*., and is similar to the true-positive rate found by Yu *et al*. This likely reflects both the higher quality of the human interactions, and also the use of the RBH method for ortholog detection. Surprisingly, despite significant combined efforts to elucidate the yeast interactome, we can still predict 405 novel protein interactions in yeast. For reasons discussed above, these interologs are largely involved in protein complexes, and help interconnect various yeast proteins and their subnetworks. This is illustrated in Additional data file 6, where the entire set of yeast predictions is shown. Black edges in this network represent interactions predicted from human that have already been shown in yeast, while the red edges represent interactions that are not contained within the current yeast interactome. To help illustrate the utility of our prediction method, we will explore in detail two complexes: the yeast replisome, and the yeast coatomer complex.

### Replisome

**Figure 4:**
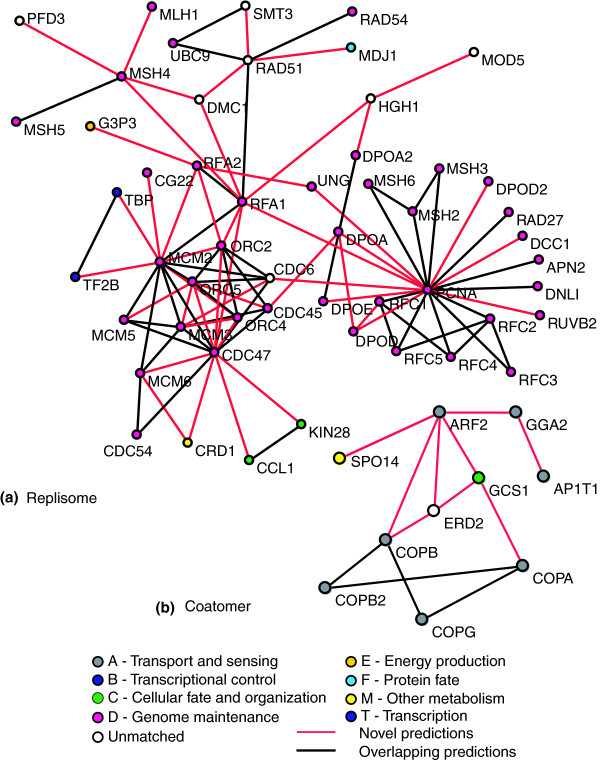
Yeast interactions transferred from the human interactome. The human interactome was used as a source to predict 750 yeast interactions, 405 of which are novel (red lines), while 345 overlap with previously known yeast PPIs. **(a) **The replisome, responsible for DNA replication, is enriched by the human interactome. **(b) **The yeast protein GCS1 is linked to retrograde transport between the Golgi and the endoplasmic reticulum through physical interactions with ERD2, ARF2, and the coatomer complex (COPA, COPB, COPB2, COPG) using human interactions. The node colors indicate the broad functional category of each protein as derived from GO annotations.

The replisome is a complex that has been extensively studied from bacteria to humans, thereby establishing the direct PPIs between many complex subunits. It has an essential role in DNA replication, as well as in DNA repair, and includes many subcomplexes, including the ORC, MCM complex, single-strand binding protein (RP-A), DNA sliding clamp (PCNA), the clamp loader (RF-C), DNA polymerases α, δ and ε, and many accessory proteins (reviewed in [38]). Figure 4a shows the replisome generated by interactions mapped from the human interactome to yeast. Some of these interactions are in the yeast interaction dataset, for example, the interactions between RFA1 and RFA2, RAD51, and MCM2. However, additional interactions, such as those involving CDC47, DMC1, HGH1, MSH4, ORC2, and PCNA, can be uniquely mapped from human. There are many other interactions among members of the ORC/MCM complexes, DNA replication components, and DNA repair components that are mapped from the human PPI network. Thus, the known human interactome, which has been generated primarily through small-scale experiments (79.4% were from LTP experiments), can be used to enrich even the yeast interactome, which has been studied extensively and systematically through multiple and technologically diverse HTP experiments.

### Coatomer complex

The coatomer protein complex is involved in the formation of vesicles that traffic between the endoplasmic reticulum (ER) and the Golgi apparatus, as well as to the plasma membrane (reviewed in [39]). Transport between these organelles is required for exporting proteins to the Golgi (anterograde transport), and recovering ER proteins from the Golgi (retrograde transport). Figure 4b illustrates some of the interactions involved in retrograde transport from the Golgi to the ER. In particular, GCS1 is a GTPase activating protein, which could conceivably activate the GTPases ARF1 and ARF2 (ARF1 not shown). ERD2 has been implicated in binding HDEL proteins, which are destined for retention in the ER. Human ERD2 has been shown to bind to ArfGAP1, the human ortholog of yeast GCS1 [40]. Both ERD2 and GCS1 interact with the COPI subunits (COPA, COPB, COPB2, and COPG), as well as the activating proteins ARF1 and ARF2. Together, these proteins control sorting and retrograde transport of HDEL-containing proteins from the Golgi to the ER. While this process has been studied extensively in yeast and humans, GCS1 has thus far only been linked to protein trafficking through genetic interactions with ARF1 and ARF2 [41]. Therefore, mapping the human PPIs to yeast suggests that GCS1 acts more specifically in the retrograde transport pathway, as opposed to anterograde transport, through its physical interaction with ERD2.

### Interolog interaction database (I2D)

To facilitate experimentation and integrated computational analysis with model organism PPI networks, we have provided all of the data discussed here in a web-accessible database [25]. This is an extension of our earlier work on OPHID [10], and covers additional target organisms. For instance, through this database the high-quality human interactome can be transferred to mouse, extending the mouse interactome by tens of thousands of protein interactions. The data are provided for download in tab-delimited text or PSI-XML format [42], and can be viewed with an OpenGL-accelerated network visualization system NAViGaTOR (Network Analysis, Visualization and Graphing, Toronto) [43] available for Windows, Linux, Solaris and OSX platforms.

## Discussion

In some respects, the human PPI dataset that we have compiled makes an ideal test set to assess the effects of interologous protein interaction prediction. For instance, due to systematic efforts at complex identification [34,44], the yeast PPI datasets are highly enriched in protein complexes. Most of the sparsely connected areas of the network are from Y2H studies, which in general have large error rates [45,46]. Thus, assessing whether the conservation of complexes across species is an artifact of experimental noise in the Y2H data or the overabundance of complexes becomes problematic. On the other hand, the sparseness of complexes in the human dataset makes it difficult to determine which types of complexes are more highly conserved: transient or stable. The analysis by Fraser [23] suggests that party hubs, or members of stable complexes, are more highly conserved. This remains to be established for human proteins, although we suspect this assertion will hold as human protein complex data become available. Additionally, the low number of complexes found in the human PPI data (Figure 1d; Additional data file 7) may have resulted in a conservative estimate for the enrichment of stable complexes in the networks created using interologs.

Clearly, care must be taken in the interpretation of PPI data analyses. Recent publications have called into question findings that were based on early versions of the yeast interactome. The correlation between high degree proteins and evolutionary rate [19,20] has been challenged by Jordan *et al*. [35], who suggest that the evolutionary conservation is instead related to highly expressed proteins in the interaction datasets. Maslov and Sneppen's [47] finding that hub-hub interactions are somehow suppressed in the interactome has been called into question by Batada *et al*. [48], a study that also concludes that 'date' and 'party' hubs [18] are artifacts of artificially small network subsets. Even the scale-free degree distribution reported for many PPI networks has been challenged [49]. These 'artifacts' have largely been attributed to inadequate sample sizes or sample bias in the early yeast PPI data. Our human PPI dataset avoids some of the sample bias that has plagued the earlier yeast data, and is analogous to the 'HC' dataset compiled by Batada [48]. Rather than being dominated by a single purification method, or HTP data alone, our human interactome is instead composed of a mix of LTP, literature-based interactions, and HTP data. This includes a variety of purification techniques, such as small-scale co-immunoprecipitations to large-scale Y2H methods.

However, the human dataset is not completely bias-free. Many of the human PPIs have been generated through LTP experiments, targeting higher abundance or disease-related proteins. This has led to a network that is more biased and sparsely connected than, for instance, the yeast interactome, which includes interactions from targeted protein complex purifications. This is exemplified in the mean degree of the human network (<k> = 6.85), compared to yeast (<k> = 33.61). The human network also has a mean clustering coefficient that is approximately half the value in yeast (<C_w_> is 0.1453 in human versus 0.2622 in yeast). While this represents a challenge in our analysis, it also highlights the need to integrate complementary interaction data to obtain more complete interactomes.

Besides showing the evolutionary conservation of the human proteins and their interactions, we were able to examine the effect on the predicted networks of interologs across species. We have shown that highly connected components of the human PPI network are more conserved than the lower degree proteins, and the proportion of proteins conserved decreases with evolutionary distance. If one is to use interologs to augment a PPI dataset, it is important to understand whether all interactions have equal probability of being transferred between organisms. In particular, signaling pathways and transient interactions (for example, kinase-substrate interactions) are of very high importance in disease processes such as cancer. It is critical, therefore, to examine the dynamic PPI networks to understand these processes. The human PPI network is a rich source of such interactions, which should survive mapping to higher eukaryotes such as mouse and rat, as nearly 70% of the human interactions are conserved in mice. For instance, using our ortholog set and examining 518 human kinases [50], 78% have an ortholog in mice, 15% and 17% have orthologs in worm and fly, respectively, while only 6% have orthologs in yeast. In contrast, 70% of the human 26S proteasome subunits have conserved orthologs in yeast, and 44% of the human RNA polymerase components are conserved in yeast. Thus, it is readily apparent that the dynamic components of the interactomes will be poorly represented in mapped networks from distantly related organisms. However, being able to transfer the wealth of protein complexes from yeast would greatly enrich the human network, which lacks information on many of the stable protein complexes that have been purified in yeast. New experimental technologies, such as the protein chip used to create the yeast kinome [31], will be required to complete the interactome within the scaffold of stable interactions that current technologies, including interolog mapping, provide.

## Materials and methods

### Datasets

The known human interactome contained in OPHID currently comprises 33,713 non-redundant PPIs, up from 16,107 when the database was first published in 2005. The network has been compiled by integrating multiple databases and experimental datasets (see Additional data file 1), and includes 9,799 proteins. The mean degree <k> in this network is 6.85, and the mean clustering coefficient <C_w_> is 0.1458.

Additional PPI datasets have been compiled for each of the model organisms. The basic characteristics of these networks are summarized in Table 1.

### Ortholog mapping

Orthologs were mapped between each of six eukaryotic organisms (*S*. *cerevisiae*, *C*. *elegans*, *D*. *melanogaster*, *M*. *musculus*, *R*. *norvegicus*, and *H*. *sapiens*) using the RBH approach as previously described [10]. Blasting was carried out on an IBM p690 mainframe using NCBI stand-alone BLAST (v.2.2.14); results were parsed using DB2 Information Integrator (v.8.1.1), and compiled in an IBM DB2 database (v.8.1.6).

### BLAST sources

BLAST sources were generated from UniProt release 7.1. Redundant Trembl sequences, which represent duplicate protein database entries, were identified and removed by blasting against organism-specific SwissProt sequences. Trembl sequences that had a SwissProt hit with e-value <1 × 10^-50 ^were flagged as redundant. Sequences shorter than 50 amino acids were ignored. The final FASTA file was constructed with all SwissProt sequences merged with the unique Trembl entries. The results of this filtering can be seen in Additional data file 8.

### Co-localization

To determine if two proteins are co-localized, a method was developed using GO terms annotating proteins in UniProt. First, primary GO terms from the cellular component (CC) aspect were retrieved for each protein from a local UniProt database (release 7.1). Terms were only included if they occurred on level 4 or greater. If any terms contained the substring 'cytosol' (for example, GO:0005842, 'cytosolic large ribosomal subunit (sensu Eukaryota)'), GO:0005737 ('cytoplasm') was added to the list. This is required because 'cytoplasm' is located at level 3 in the GO tree, along with many other very general terms. Next, all parent terms were added to the annotation lists provided that the parents were from level 5 or below. Finally, if any terms were found in the intersection of the two GO term lists, the proteins were marked as co-localized. While this method is very stringent and comes at the expense of a higher false negative rate on co-localizations, it avoids considering two proteins as co-localized with only very general annotations, and is fully automated.

### Clustering coefficient (C_w_)

The clustering coefficient was introduced to measure if the network has small-world properties [51]. C_w _measures the proportion of edges between the nodes within its neighbourhood divided by the number of edges that could possibly exist between them:

Cw=2⋅eijkw(kw−1)
 MathType@MTEF@5@5@+=feaafiart1ev1aaatCvAUfeBSjuyZL2yd9gzLbvyNv2Caerbhv2BYDwAHbqedmvETj2BSbqee0evGueE0jxyaibaiKI8=vI8tuQ8FMI8Gi=hEeeu0xXdbba9frFj0=OqFfea0dXdd9vqai=hGuQ8kuc9pgc9s8qqaq=dirpe0xb9q8qiLsFr0=vr0=vr0dc8meaabaqaciGacaGaaeqabaqadeqadaaakeaacaWGdbWaaSbaaSqaaiaadEhaaeqaaOGaeyypa0ZaaSaaaeaacaaIYaGaeyyXICTaamyzamaaBaaaleaacaWGPbGaamOAaaqabaaakeaacaWGRbWaaSbaaSqaaiaadEhaaeqaaOGaaiikaiaadUgadaWgaaWcbaGaam4DaaqabaGccqGHsislcaaIXaGaaiykaaaaaaa@4385@

where e_ij _is the number of edges between all neighbors *i *and *j *of node *w*, k_*w *_is the degree of node *w*, and k_*w*_(k_*w *_- 1) is the number of possible edges in the neighborhood of node *w*. The mean C_w _(<C_w_>) was computed over all nodes with k_w _> 1.

## Additional data files

The following additional data are available with the online version of this paper. Additional data file 1 contains a list of all the PPI datasets that were compiled and used in this study, along with their sources. Additional data file 2 lists the properties of the source and predicted protein interaction networks, including overlapping PPI, clustering coefficient (C_w_), and average protein degree (<k>). Additional data file 3 shows the high confidence subset of yeast PPI [28] data, integrated with gene expression data from Gasch *et al*. [29]. Additional data file 4 shows the cumulative distributions of joint sequence identity [4] for PPI mapped from humans to the model organisms. Additional data file 5 contains results of permutation testing on co-localization of protein pairs. Additional data file 6 shows the overlap between the yeast PPI network, and the predictions made from the human interactome. Additional data file 7 shows the yeast PPI network constructed using predictions from human PPIs, illustrating the conservation of protein complexes. Additional data file 8 lists the results of filtering the BLAST data sources for redundant protein sequences.

## Supplementary Material

Additional data file 1 PPI datasets that were compiled and used in this study, along with their sources. Click here for file

Additional data file 2 Properties of the source and predicted protein interaction networks, including overlapping PPI, clustering coefficient (C_w_), and average protein degree (<k>). Click here for file

Additional data file 3 High confidence subset of yeast PPI [28] data, integrated with gene expression data from Gasch *et al*. [29]. Click here for file

Additional data file 4 Cumulative distributions of joint sequence identity [4] for PPI mapped from humans to the model organisms. Click here for file

Additional data file 5 Results of permutation testing on co-localization of protein pairs. Click here for file

Additional data file 6 Overlap between the yeast PPI network, and the predictions made from the human interactome. Click here for file

Additional data file 7 Yeast PPI network constructed using predictions from human PPIs, illustrating the conservation of protein complexes. Click here for file

Additional data file 8 Results of filtering the BLAST data sources for redundant protein sequences. Click here for file
