# Supplementary figures and images for: Unequal evolutionary conservation of human protein interactions in interologous networks
Source: Genome Biol. 2007 May 29;8(5):R95. doi: 10.1186/gb-2007-8-5-r95 (PMC1929159; doi:10.1186/gb-2007-8-5-r95)

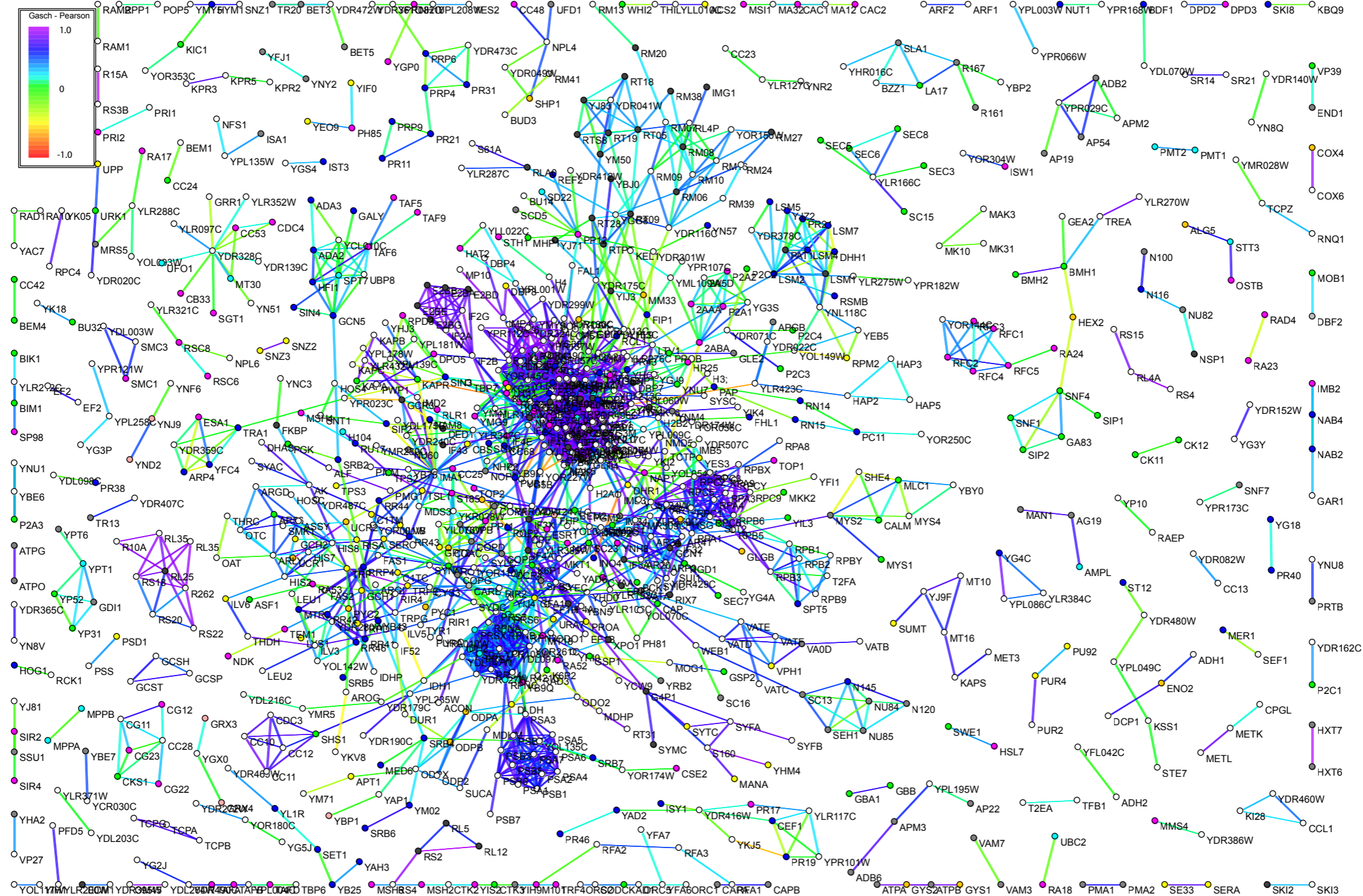

Supplement: Additional data file 3 — High confidence subset of yeast PPI [28] data, integrated with gene expression data from Gasch et al. [29]. [file gb-2007-8-5-r95-S3.pdf]

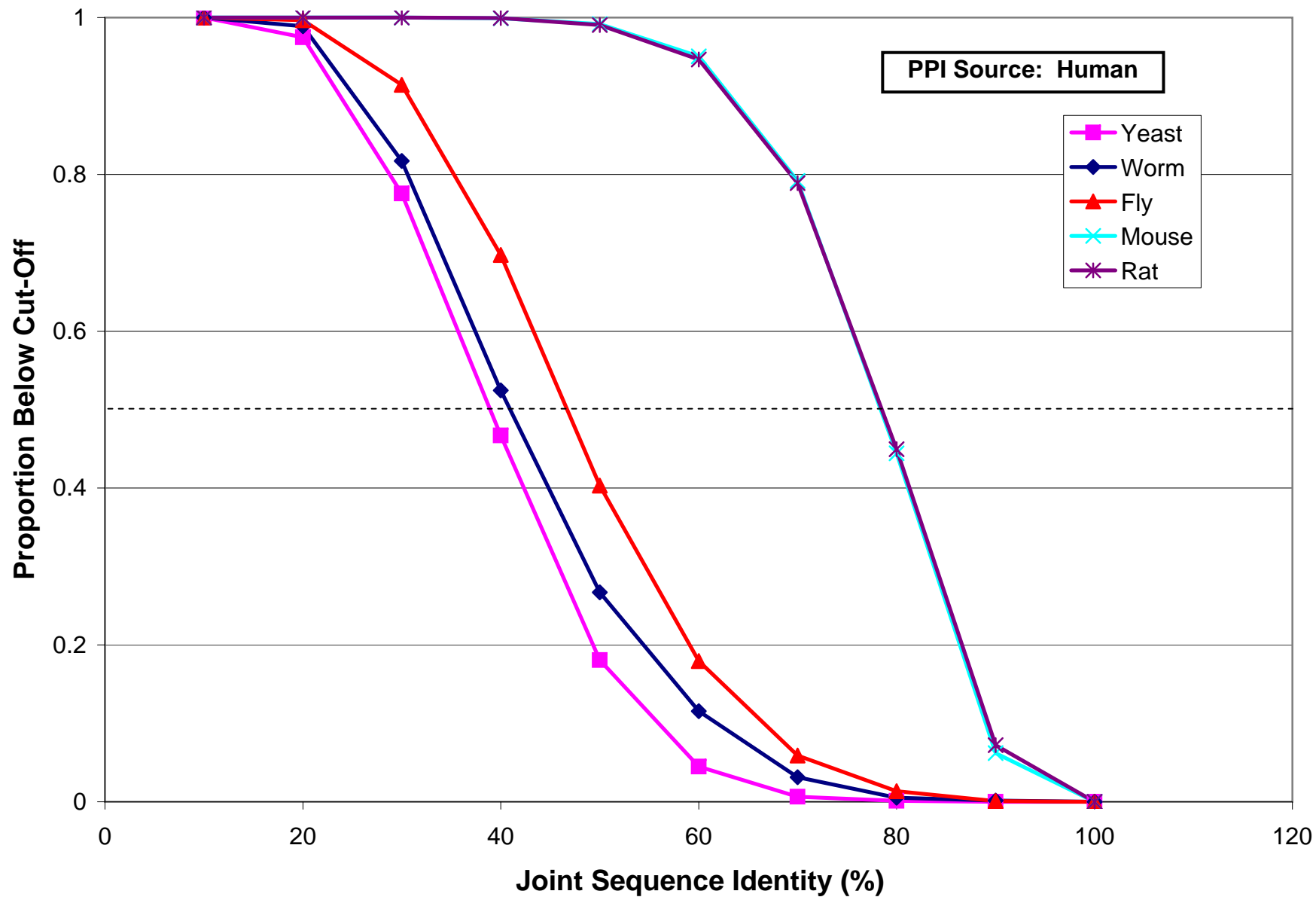

Supplement: Additional data file 4 — Cumulative distributions of joint sequence identity [4] for PPI mapped from humans to the model organisms. [file gb-2007-8-5-r95-S4.pdf]

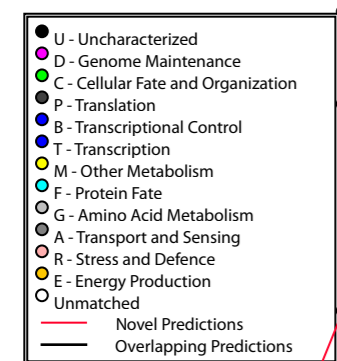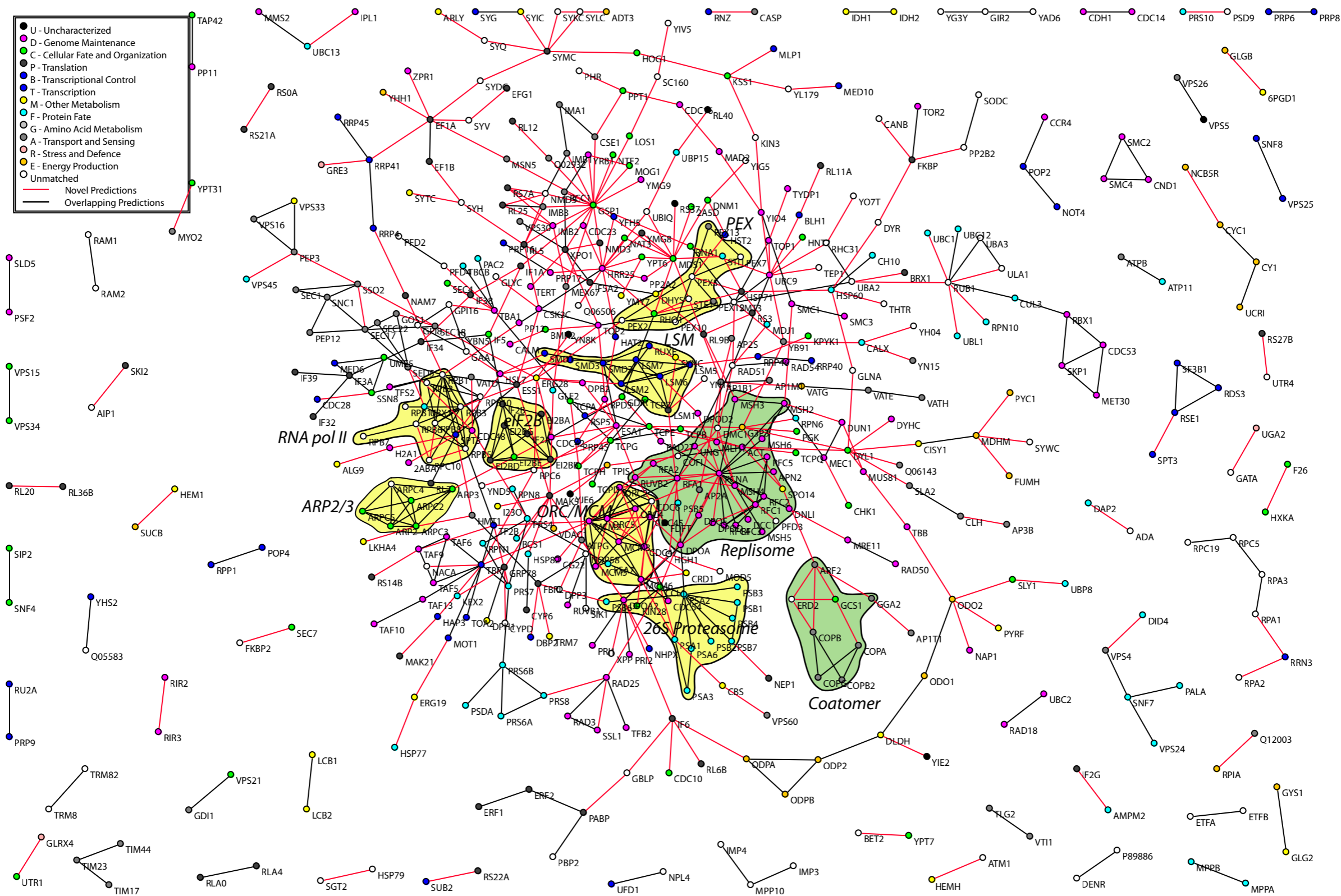

Supplement: Additional data file 6 — Overlap between the yeast PPI network, and the predictions made from the human interactome. [file gb-2007-8-5-r95-S6.pdf]

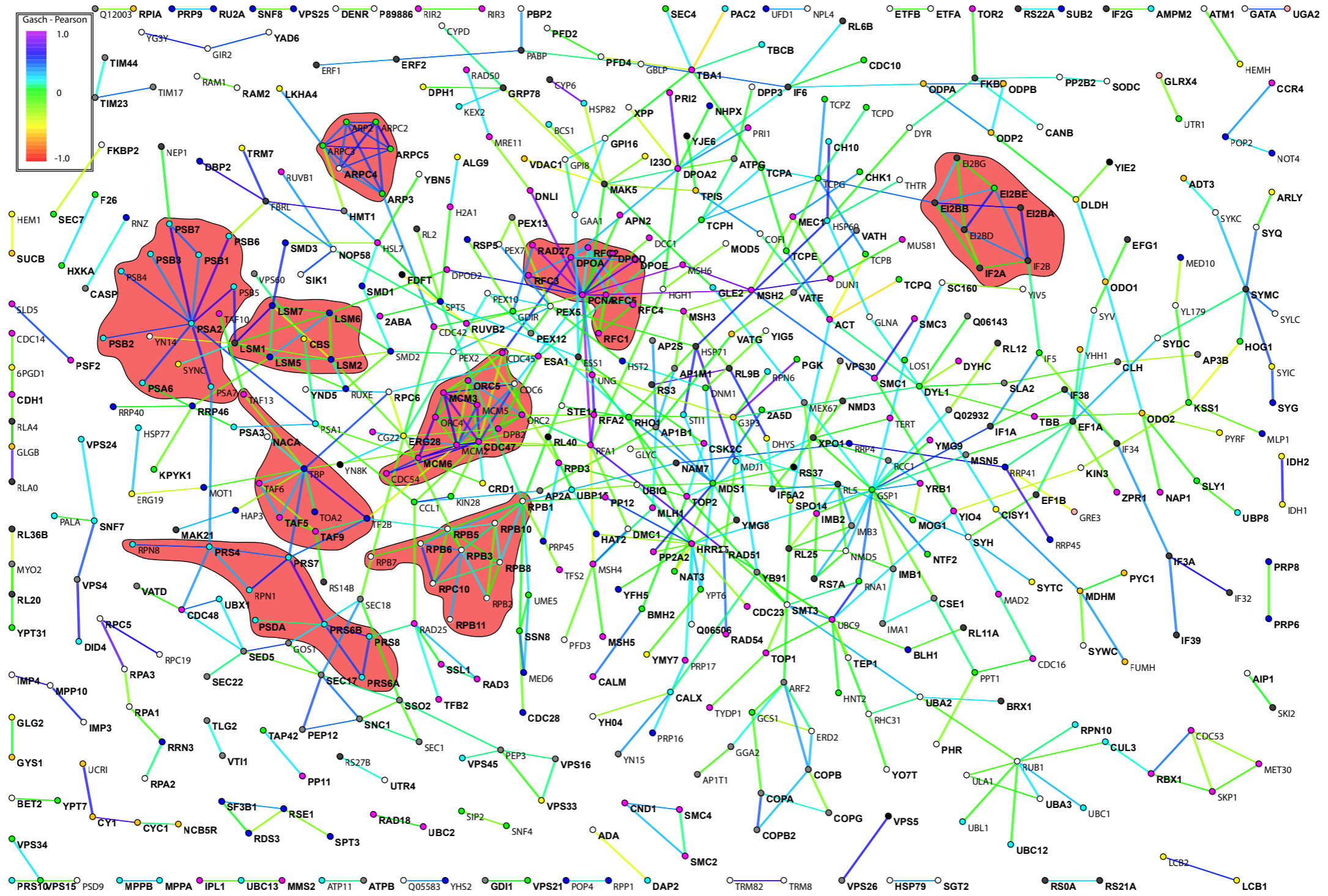

Supplement: Additional data file 7 — Yeast PPI network constructed using predictions from human PPIs, illustrating the conservation of protein complexes. [file gb-2007-8-5-r95-S7.pdf]
