# Supplementary material for: Unequal evolutionary conservation of human protein interactions in interologous networks
Source: Genome Biol. 2007 May 29;8(5):R95. doi: 10.1186/gb-2007-8-5-r95 (PMC1929159; doi:10.1186/gb-2007-8-5-r95)
Supplement: Additional data file 5 — Results of permutation testing on co-localization of protein pairs. [file gb-2007-8-5-r95-S5.doc]

## Permutation Analysis of Co-Localization

Logically, proteins must be co-localized in order to physically interact. However, in practice, the annotation of protein sub-cellular localization is far from complete. We have attempted to control for differences in the level of annotation of the interacting proteins by comparing only those proteins that have at least one entry for sub-cellular localization, and will expand on this analysis here.

Figure S5 shows the results of permutation tests that were done to compare the background levels of co-localization within each organism. Not surprisingly, the yeast proteins have the largest fraction of proteins with localization data. This would be expected based on the studies in yeast that have localized 75% of the predicted ORFs [51, 52]. However, proteins in multi-cellular organisms have not been as well studied. Importantly, while *C. elegans* has the highest level of co-localization in our mapped networks (Figure 3B), it actually has the lowest level of annotation of the 6 organisms tested. This indicates that it is neither a bias in the annotation of any one organism, nor random chance that produces the patterns that we see in our mapped networks. We further show that the level of co-localization in each experimentally derived PPI dataset (EXP; red bars) is similar, ranging from 43 – 66%.

In order to show that the enrichment in co-localization in our mapped networks is not an artifact of the background level of annotation, we performed a permutation analysis as follows. For each organism, we chose 10,000 random protein pairs with replacement from the set of all proteins in SwissProt/Trembl that have sub-cellular localization data (yellow bars). In the random protein pairs, 29% of yeast proteins are co-localized, while only 18 – 23% of the other organisms have co-localized pairs. Thus, there is some bias due to the higher level of annotation of yeast proteins, but the value that we obtained in the mapped network (70.4%) is significantly higher than the random pairs

(p = 1.78 x 10^-98^, Fisher’s exact test). This is also true for the other organisms.

**Figure S5 – Co-localization of Protein Pairs in Permuted Datasets.** In order to show that the increased co-localization is not due to random chance, or biases in the annotation of the proteins in each organism, we generated sets of 10,000 random proteins pairs. (blue bars) The fraction of all proteins in each organism that have GO sub-cellular localization data in SwissProt. (red bars) The fraction of the experimental interactomes with co-localized protein pairs. (yellow bars) Randomly selected protein pairs from the set of all SwissProt proteins that have localization data. (light blue bars) Randomly selected protein pairs from the set of SwissProt proteins that comprise the experimental interactomes (i.e., the interactomes themselves were permuted). (purple bars) Randomly selected protein pairs from the set of proteins that comprise the interactomes and have orthologs in humans.

In order to show that our results are not due to increased annotation of proteins that are involved in the PPI networks, we performed the same permutation tests after restricting the protein set from which we randomly sampled to the proteins within the interactomes that had localization data (light blue bars). The trends were similar to the previous analysis, suggesting that a bias in annotation for proteins that are better characterized is not a factor.

Finally, to rule out any bias due to increased annotations for ancient, highly conserved proteins, we filtered the protein pool further, restricting it to proteins that had orthologs in humans (purple bars). Once again, we can see that pairs chosen randomly from this set have a relatively low level of co-localization. Most importantly, these results show that the randomly selected pairs do not follow the trends observed in Figure 3B; that is, the random set from the worm has the second lowest level of co-localization (14.5%), while it is the highest in our mapped networks (Figure 3B).

In regards to co-localization being a feature of protein complexes, we have noted a general trend in the interaction data on this in several of our analyses. As an example, 48.1% of the experimentally-derived yeast PPIs are co-localized, which is similar to the other interactomes. If we analyze the yeast ‘high confidence’ dataset from von Mering *et al*. [28] we find that 85.7% of the PPIs (n = 1,601) with annotations for both proteins are co-localized. This particular dataset is enriched for complexes due to the overlapping of 5 – 6 different techniques, thereby detecting strong, stable interactions. In another dataset, the new TAP tagging dataset [36] has a co-localization of 88.3% (n = 6,705). The very nature of the TAP tagging approach enriches for protein complexes. This is in contrast to the dataset of Ptacek *et al.* [31] that comprises transient kinase:substrate interactions, where only 36.4% of the PPIs are co-localized.

We have shown here using permuted datasets that the trends we observed in our analysis are not due to random chance. Furthermore, our observations do not appear to stem from the differences in annotation in any one organism. Finally, our analysis of complex-enriched vs. transient-rich datasets demonstrates that complexes display higher co-localization in Gene Ontology annotations.

## References

1. Huh WK, Falvo JV, Gerke LC, Carroll AS, Howson RW, Weissman JS, O'Shea EK: **Global analysis of protein localization in budding yeast.** *Nature* 2003, **425:**686-691.

2. Kumar A, Agarwal S, Heyman JA, Matson S, Heidtman M, Piccirillo S, Umansky L, Drawid A, Jansen R, Liu Y, et al: **Subcellular localization of the yeast proteome.** *Genes Dev* 2002, **16:**707-719.

3. von Mering C, Krause R, Snel B, Cornell M, Oliver SG, Fields S, Bork P: **Comparative assessment of large-scale data sets of protein-protein interactions.** *Nature* 2002, **417:**399-403.

4. Collins SR, Kemmeren P, Zhao XC, Greenblatt JF, Spencer F, Holstege FC, Weissman JS, Krogan NJ: **Towards a comprehensive atlas of the physical interactome of Saccharomyces cerevisiae.** *Mol Cell Proteomics* 2007.

5. Ptacek J, Devgan G, Michaud G, Zhu H, Zhu X, Fasolo J, Guo H, Jona G, Breitkreutz A, Sopko R, et al: **Global analysis of protein phosphorylation in yeast.** *Nature* 2005, **438:**679-684.
